# Deficit irrigation and fertilization strategies to improve soil quality and alfalfa yield in arid and semi-arid areas of northern China

**DOI:** 10.7717/peerj.4410

**Published:** 2018-02-21

**Authors:** Qianmin Jia, Muhammad Kamran, Shahzad Ali, Lefeng Sun, Peng Zhang, Xiaolong Ren, Zhikuan Jia

**Affiliations:** 1 Northwest A&F University, Institute of Water Saving Agriculture in Arid Areas of China, Yangling, Shaanxi, China; 2 Northwest A&F University, Key Laboratory of Crop Physi-ecology and Tillage Science in North-western Loess Plateau, Ministry of Agriculture, Yangling, Shaanxi, China; 3 Northwest A&F University, College of Forestry, Yangling, Shaanxi, China; 4 Northwest A&F University, College of Agronomy, Yangling, Shaanxi, China

**Keywords:** Soil organic carbon, Microbial biomass, Deficit irrigation, Soil respiration rate, Soil enzyme

## Abstract

**Background:**

In the arid and semi-arid areas of northern China, overexploitation of fertilizers and extensive irrigation with brackish groundwater have led to soil degradation and large areas of farmland have been abandoned. In order to improve the soil quality of abandoned farmland and make reasonable use of brackish groundwater, we conducted field trials in 2013 and 2014.

**Methods:**

In our study, we used three fertilization modes (CF, chemical fertilizer; OM, organic manure and chemical fertilizer; NF, no fertilizer) and three deficit irrigation levels (I_0_: 0 mm; I_75_: 75 mm; I_150_: 150 mm).

**Results:**

The results showed that the activities of soil urease, alkaline phosphatase, invertase, catalase, and dehydrogenase in the OM treatment were significantly improved compared with those in the CF and NF treatments under the three deficit irrigation levels. Compared with NF, the OM treatment significantly increased soil organic carbon (SOC), water-soluble carbon (WSC), total nitrogen, microbial biomass carbon and nitrogen (MBC and MBN), and soil respiration rate, and significantly decreased soil C:N and MBC:MBN ratios and the metabolic quotient, thus improving the soil quality of abandoned farmland. Furthermore, the OM treatment increased alfalfa plant height, leaf area index, leaf chlorophyll content, and biomass yield. Under the CF and OM fertilization modes, the activities of urease and catalase in I_150_ were significantly higher than those in I_0_, whereas irrigating without fertilizer did not significantly increase the activity of these two enzymes. Regardless of fertilization, alkaline phosphatase activity increased with an increase in irrigation amount, whereas invertase activity decreased.

**Discussion:**

The results showed that deficit irrigation with brackish groundwater under the OM treatment can improve soil quality. Over the two years of the study, maximum SOC, total nitrogen, WSC, MBC, and MBN were observed under the OM-I_150_ treatment, and the alfalfa biomass yield of this treatment was also significantly higher than that of the OM-I_0_ treatment. Therefore, the OM-I_150_ treatment could be used as a suitable measure not only to improve the quality of abandoned farmland soil but also to increase the alfalfa biomass yield in arid and semi-arid areas of northern China.

## Introduction

Exploitation and utilization of brackish groundwater has become a concern in many countries (Oster, 1994; Mondal, Bhuiyan & Franco, 2001; Jiang et al., 2012). Many agricultural irrigation studies have shown that rational use of brackish groundwater does not result in a reduction of crop yields (Wang et al., 2007; Kang, Chen & Wan, 2010). However, prolonged periods of brackish water irrigation have been found to increase soil salinity, resulting in the destruction of soil structural stability (Lax et al., 1994), in addition to reduced microbial biomass and soil enzyme activity (Rietz & Haynes, 2003), resulting in severe soil degradation (Romic et al., 2008; Wang et al., 2016). In the arid and semi-arid areas of China, natural precipitation cannot meet the needs of crop growth, due to the shortage of fresh water resources on the surface, and therefore farmers mainly rely on the use of brackish groundwater for irrigation purposes (Kang, Chen & Wan, 2010; Jiang et al., 2012). Although brackish water can be used to provide more water for crops, it also introduces large amounts of salt into the soil, resulting in large areas of soil secondary salinization, and as a consequence, many farmlands have been abandoned (Pang et al., 2010; Bouksila et al., 2013). Alfalfa (*Medicago sativa* L.), as a good legume forage, not only feeds livestock but also improves soil quality (Raiesi, 2007; Yong, 2007), and is widely grown in many countries (Raiesi, 2007; Li & Huang, 2008; Bagavathiannan, Gulden & Acker, 2011). Alfalfa is cultivated over more than 4 ×10^6^ hm^2^ in China, mainly in the arid and semi-arid regions of northern China (Jing et al., 2016); however, large areas of alfalfa in these area need irrigation to maintain normal growth and development. Therefore, it is important to study rational irrigation patterns to increase alfalfa production and prevent land degradation. In recent years, deficit irrigation has been widely used in agricultural production to improve crop yield and water-use efficiency (Shock et al., 2007; Du et al., 2010; Lindenmayer et al., 2011). However, it is not known whether using brackish water for deficit irrigation can improve soil quality and increase alfalfa production, and thus this needs further study.

It is well known that chemical fertilizers can be used to increase soil nutrients and that their application is a major approach for increasing crop yields (Verma & Sharma, 2007; Moharana et al., 2012). However, overapplication of chemical fertilizers has resulted in lower soil organic matter content and decreased soil enzyme activities and microbial populations (Saha et al., 2008), which in turn has caused severe soil degradation. The level of soil enzyme activity reflects the biological activity and biochemical reactions in the soil and is an important index used to evaluate soil health (Li et al., 2010). Soil microorganisms play an important role in the decomposition of organic matter, altering soil enzyme activity and nutrient cycling (Bastida et al., 2008; Saha et al., 2008). Most studies have shown that organic manure is beneficial to soil microbial growth (Hao et al., 2008; Liu et al., 2010) and can improve soil organic carbon content and enzyme activity (Liang et al., 2005; Yang et al., 2008), thereby improving the soil environment. However, compared with chemical fertilizers, the available nutrients in organic manures are low, which is not beneficial for crops to absorb and utilize nutrients in a short time (Marinari et al., 2000; Hao et al., 2008). The use of organic manure alone leads to a decrease in the yield of some crops (Enujeke & Egbuchua, 2013; Xin et al., 2017). However, some studies have shown that a combination of organic manure and chemical fertilizer is a more effective means of fertilization, which can significantly increase soil available nutrient content, soil enzyme activity, and soil microbial biomass carbon and nitrogen (Qiu et al., 2004; Xin et al., 2017), and also modify soil respiration rate (Bastida et al., 2008; Hao et al., 2008), thus increasing the crop yield while maintaining a good soil environment (Moharana et al., 2012). Although many studies have demonstrated the utility of applying organic manure in combination with chemical fertilizers, it is necessary to study how to use deficit irrigation with brackish water under combined fertilization conditions.

In order to improve soil quality in abandoned farmland and increase pasture production, we studied the effects of three fertilization modes and different deficit irrigation rates on soil enzyme activities, organic carbon, water-soluble carbon, total nitrogen, microbial biomass, respiration rate, and alfalfa yield. The purpose is to determine the appropriate fertilization methods and the irrigation volume of brackish groundwater. We believe that an appropriate combination of organic manure and chemical fertilizer with deficit irrigation can improve the soil environment and alfalfa yield in arid and semi-arid areas of northern China.

## Materials and Methods

### Study site and materials

**Figure 1:**
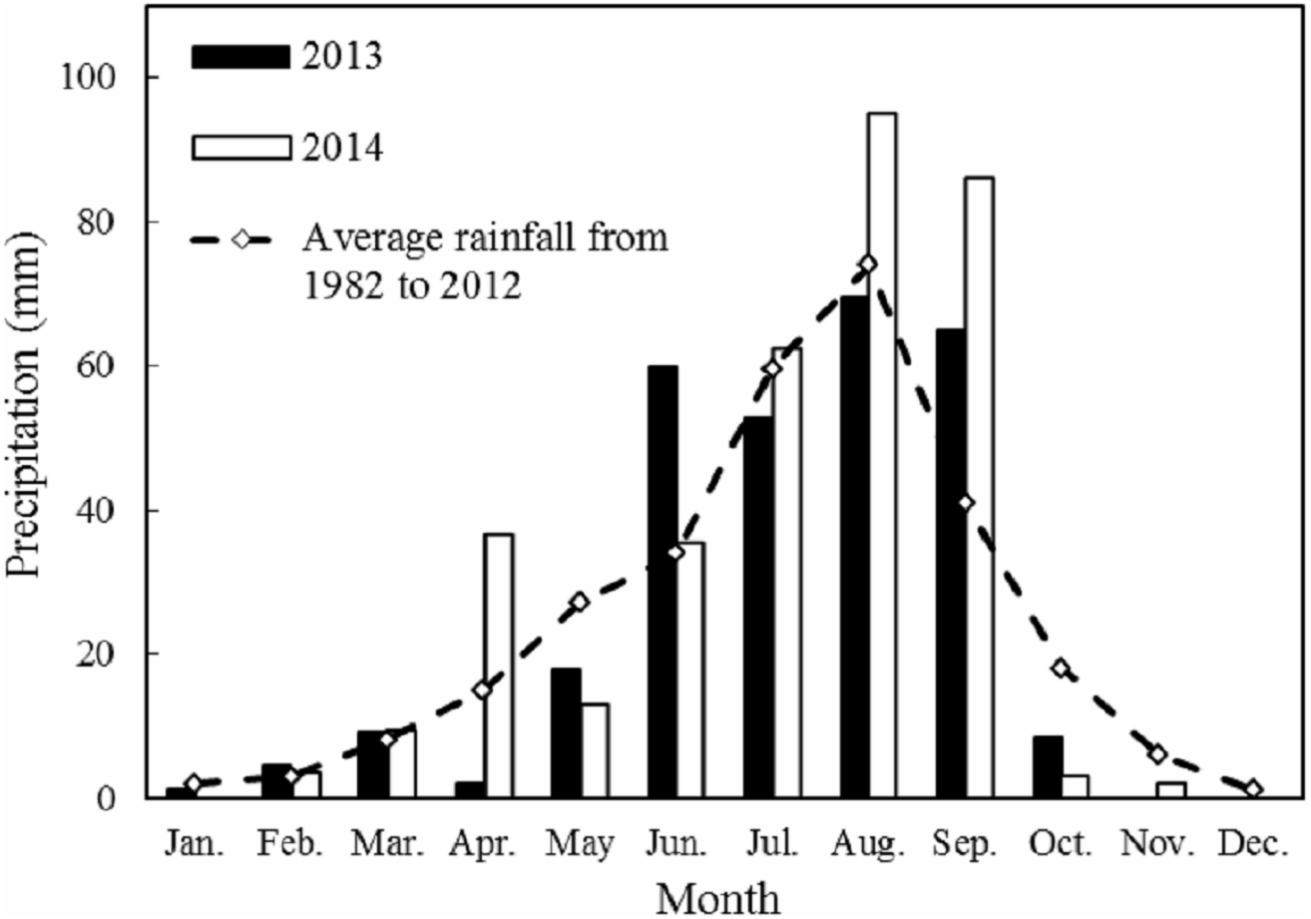
Monthly rainfall in 2013 and 2014 with the 31-year average (1982–2012) at the Yanchi Experimental Station, Ningxia Province, China.

Field studies were performed in 2013 and 2014 in Yanchi County, NingXia (106°30′–107°47′E, 37°04′–38°10′N) on the Loess Plateau, China. The regional climate is a temperate continental climate. The average annual temperature is 7.7 °C, with extremes ranging from a high of 38.1 °C to a low of −29.6 °C. The annual precipitation rate over last 31 years is 288.6 mm and the average annual potential evaporation is 2,710 mm. The amount of rainfall was 290.4 mm in 2013 and 346.9 mm in 2014. The monthly rainfall amounts during these 2 years and the 31-year monthly averages (1982–2012) are shown in Fig. 1. The study area was farmland that has been abandoned for 11 years and has abundant reserves of brackish groundwater (NaCl: 2.3 g L^−1^). The soil characteristics of the 0–20 cm soil layer in 2012 were as follows: soil organic carbon, 3.71 g kg^−1^; total nitrogen (N), 0.28 g kg^−1^; total phosphorus (P), 0.21 g kg^−1^; total potassium (K), 15.52 g kg^−1^; available N, 34.11 mg kg^−1^; available P, 3.62 mg kg^−1^; available K, 100.58 mg kg^−1^; and total salt content, 2.08 g kg^−1^, with a pH of 8.36. Herdsmen in the area process large amounts of sheep manure into organic fertilizer and the organic fertilizers we used contained 186.5 g kg^−1^ of organic C, 7.1 g kg^−1^ of total N, 4.5 g kg^−1^ of total P, and 13.2 g kg^−1^ of total K. Chemical fertilizer N was supplied as urea (N 46%), P was supplied as calcium superphosphate (P_2_O_5_ 20%), and K was supplied as potassium sulphate (K_2_O 50%).

### Experimental design and field management

The experiment was laid out in randomized complete block design (RCBD) and included three different fertilization methods and three deficit irrigation rates. The fertilization methods were (i) chemical fertilizer (CF: N 65, P 180, and K 260 kg ha^−1^), (ii) organic manure + chemical fertilizer (OM: organic manure 12,000 kg ha^−1^ + P 126 kg ha^−1^ and K 102 kg ha^−1^), and (iii) no fertilization (NF). Each fertilization method included the following three deficit irrigations rates: 0 mm (I_0_), 75 mm (I_75_), and 150 mm (I_150_). Each treatment was performed in triplicate, with a combined total of 27 plots, each of 30 m^2^ (5 m × 6 m). CF and OM treatments were applied with a total N of 65 kg ha^−1^ (including inorganic and organic N), total P of 180 kg ha^−1^, and total K of 260 kg ha^−1^. We used a small ploughing machine to bring the fertilizer into the surface soil. Organic manure and chemical fertilizer were used as the base fertilizer and ploughed into the soil layer before sowing alfalfa (2 April 2013) and at the re-greening stage (8 April 2014). Alfalfa (*Medicago sativa* L. cultivar Zhongmu No. 1) seeds were sown on 15 April 2013, at a seeding rate of 30 kg ha^−1^, and plants were spaced at 30 cm intervals. Half of the total irrigation was applied (i.e., OM_75_ and OM_150_ treatments were 37.5 and 75 mm, respectively) after sowing alfalfa in 2013 (16 April) and at the re-greening stage in 2014 (9 April), and the other half was applied at the branching stage (26 May 2013 and 28 May 2014).

### Data collection

At the alfalfa flowering stage (22 June 2013; 25 June 2014), nine alfalfa plants were randomly selected in each plot to measure plant height with a ruler. The relative chlorophyll content (SPAD value) of six leaves in the middle of each plant was determined using a SPAD 502 Chlorophyll Meter (Konica Minolta Inc., Osaka, Japan), and the average value was calculated as the SPAD value per plant. Leaf area index (LAI) was measured using a photosynthetically active radiation ceptometer (AccuPAR model LP-80; Decagon Devices, Pullman, WA, USA), which was placed parallel to the row direction and near the alfalfa roots of each plot in three different positions. At the alfalfa flowering stage (23 June 2013; 26 June 2014), three sub-plots of 1 m^2^ in each plot were selected to harvest the alfalfa. The plants were placed in an oven at 70 °C for at least 48 h and dried to constant weight to obtain aboveground biomass.

After the alfalfa harvest (25 June 2013; 27 June 2014), soil samples were collected from each plot at depths of 0–20 cm. The soil samples were collected from five points in each plot replicate and mixed to produce a composite sample. A portion of this composite sample was used for the determination of soil microbial biomass and soil respiration rate. The remaining portion of the soil sample was placed in a sterile preservation bag and stored immediately at 4 °C for the quantification of soil enzymatic activities (soil urease, alkaline phosphatase, invertase, catalase, and dehydrogenase). The soil urease activity was determined using a phenol-sodium hypochlorite colorimetric method as described by Li et al. (2010). Alkaline phosphatase activity was determined according to the procedure of Tabatabai (1994), whereas invertase activity was determined by colorimetric analysis using 3,5-dinitrosalicylic acid (Frankeberger & Johanson, 1983). Catalase activity was determined by the residual H_2_O_2_ back-titration method following Li et al. (2010), and the activity of dehydrogenase was based on the method of García et al. (1993).

Soil microbial biomass carbon (MBC) was determined using the fumigation-extraction method as described by Vance, Brookes & Jenkinson (1987), and soil microbial biomass nitrogen (MBN) was determined using the method described by the Brookes et al. (1985). Soil respiration rate was determined by the method of Wildung, Garland & Buschbom (1975). The metabolic quotient (qCO_2_) was determined according to the method of Anderson & Domsch (1993). The qCO_2_ represents the ratio between microbial biomass carbon and soil organic carbon. Soil organic carbon (SOC) was measured following the wet oxidation method using K_2_Cr_2_O_7_ digestion with H_2_SO_4_ and H_3_PO_4_ as described by Snyder & Trofymow (1984). Total nitrogen (TN) was measured using the semi-macro Kjeldahal method (i.e., by titration with H_2_SO_4_). Water-soluble carbon (WSC) was measured using the wet oxidation method described by Sims & Haby (1971).

### Statistical analysis

The data were analysed using a residual test method prior to statistical analysis, and the data met the assumption of homogeneity of variances and followed normal distribution. The experimental data were analysed using SPSS 22.0 (SPSS Inc. Chicago, IL, USA). The data from each sampling event were analysed separately. The means among treatments were compared based on the least significant difference test (LSD 0.05).

## Results

### Soil enzyme activities

The results of the variance analysis showed that the effects of fertilization (F) and irrigation (I) on urease, invertase, and catalase activity were highly significant (*P* < 0.01) in the 2-year experiment (Table 1). Furthermore, the effect of F on alkaline phosphatase and dehydrogenase was highly significant (*P* < 0.01), whereas the effect of I on dehydrogenase was not significant (*P* > 0.05).

**Table 1:** Effects of treatments^a^ on urease, alkaline phosphatase, invertase, catalase, and dehydrogenase activities in 2013 and 2014^b^.

Year	Fertilization	Irrigation	Alkaline phosphatase (mg PNP kg^−1^ h^−1^)	Invertase (mg g^−1^24 h^−1^)	Urease (mg NH3 kg^−1^ h^−1^)	Catalase (0.1 mol l^−1^ KMnO_4_ ml g^−1^)	Dehydrogenase (mg TPF kg^−1^ 24 h^−1^)
2013	CF	I_0_	255 ± 23de	9.0 ± 0.5bc	6.8 ± 0.4d	0.8 ± 0.1c	53.3 ± 6.0c
		I_75_	285 ± 25cd	7.9 ± 0.6cd	7.6 ± 0.4cd	0.9 ± 0.1bc	54.9 ± 7.6bc
		I_150_	310 ± 28bc	7.0 ± 0.6de	8.0 ± 0.4c	1.0 ± 0.1b	56.8 ± 7.3bc
	OM	I_0_	310 ± 36bc	10.6 ± 0.9a	8.4 ± 0.6bc	1.0 ± 0.2b	69.0 ± 9.6ab
		I_75_	349 ± 35ab	9.8 ± 1.0ab	9.2 ± 0.7ab	1.5 ± 0.2a	73.8 ± 11.9a
		I_150_	366 ± 40a	9.0 ± 0.9bc	9.8 ± 0.9a	1.7 ± 0.3a	76.6 ± 13.2a
	NF	I_0_	202 ± 14f	6.0 ± 0.3ef	3.4 ± 0.2e	0.5 ± 0.0d	47.3 ± 3.8c
		I_75_	231 ± 13ef	5.4 ± 0.3f	3.8 ± 0.3e	0.6 ± 0.1d	46.5 ± 4.3c
		I_150_	255 ± 12de	5.0 ± 0.3f	4.0 ± 0.2e	0.7 ± 0.1cd	47.7 ± 4.0c
	ANOVA	F	^**^	^**^	^**^	^**^	^**^
		I	^*^	^**^	^**^	^**^	ns
		F × I	ns	ns	ns	ns	ns
2014	CF	I_0_	291 ± 19de	11.4 ± 0.8b	7.0 ± 0.4e	1.1 ± 0.2cd	52.8 ± 7.0d
		I_75_	320 ± 21cd	10.6 ± 0.9bc	7.9 ± 0.5de	1.4 ± 0.2bc	53.4 ± 7.3d
		I_150_	342 ± 23bc	9.1 ± 0.7cd	8.3 ± 0.6cd	1.5 ± 0.2b	56.4 ± 6.2cd
	OM	I_0_	336 ± 30bc	14.5 ± 1.4a	9.1 ± 0.7bc	1.5 ± 0.3b	79.0 ± 10.4ab
		I_75_	371 ± 34ab	13.1 ± 1.4a	9.6 ± 0.8ab	1.6 ± 0.2ab	86.6 ± 16.2ab
		I_150_	399 ± 41a	11.2 ± 1.4b	10.2 ± 0.8a	1.9 ± 0.3a	89.0 ± 14.4a
	NF	I_0_	230 ± 13f	9.1 ± 0.5cd	3.7 ± 0.2f	0.7 ± 0.0e	46.3 ± 2.6d
		I_75_	256 ± 10ef	8.5 ± 0.5de	4.0 ± 0.3f	0.8 ± 0.1de	45.3 ± 3.8d
		I_150_	269 ± 15de	7.3 ± 0.5e	4.4 ± 0.4f	1.0 ± 0.1de	50.2 ± 4.0d
	ANOVA	F	^**^	^**^	^**^	^**^	^**^
		I	^*^	^**^	^**^	^**^	ns
		F × I	ns	ns	ns	ns	ns

**Notes.**

a CF, Chemical fertilizer; OM, Organic manure and chemical fertilizer; NF, No fertilizer; I_0_, 0 mm irrigation; I_75_, 75 mm irrigation; I_150_, 150 mm irrigation; F, fertilization; I, Irrigation; ANOVA, analysis of variance; ns, Not significant.

b Values are given as means ± standard deviation, and different lowercase letters indicate significant differences at the 5% probability level (LSD; *n* = 3).

* Significant at 5% probability level.

** Significant at 1% probability level.

Under the three fertilization modes in the two years, alkaline phosphatase activity in the I_150_ treatment was significantly higher than that in the I_0_ treatment, whereas invertase activity was significantly lower than in the I_0_ treatment, and there was no significant difference in dehydrogenase activity between the different irrigation treatments. In the CF and OM patterns, the urease and catalase activities in the I_150_ treatment were significantly higher than those in the I_0_ treatment. However, in the NF patterns, there was no significant difference in urease and catalase activities between each irrigation treatment. Our research indicated that brackish groundwater has a less inhibitory effect on soil enzyme activity due to the lower salt content (NaCl: 2.3 g L^−1^) in the water. The urease and catalase activities can be increased by using brackish water under fertilization conditions.

The two-year averages show that, compared with the NF treatment, the urease, alkaline phosphatase, invertase, catalase, and dehydrogenase activities in the OM treatment increased by 5.5 mg NH_3_ kg^−1^ h^−1^ (144%), 114.7 mg PNP kg^−1^ h^−1^ (48%), 4.5 mg g^−1^24 h^−1^ (66%), 0.8 0.1 mol l^−1^ KMnO_4_ mL g^−1^ (120%), and 30.6 mg TPF kg^−1^ 24 h^−1^(65%), respectively. Under the same irrigation pattern, the activities of the five enzymes in the OM treatment were significantly higher than those in the CF and NF treatments. Organic manure combined with chemical fertilizers can significantly increase soil enzyme activity, particularly under conditions of deficit irrigation. The two-year averages show that the highest alkaline phosphatase, urease, catalase and dehydrogenase activities were observed under the OM-I_150_ treatment. This is because the irrigation amount (150 mm) under the OM-I_150_ treatment is lower than the conventional irrigation (300 mm) in the area, and thus soil enzyme activities are less inhibited by salt stress.

### Soil organic carbon (SOC), water-soluble carbon (WSC), total nitrogen (TN), and the SOC: TN ratio

Variance analysis indicated that the effects of F on SOC, TN, the SOC:TN ratio, and WSC were highly significant (*P* < 0.01) in the two-year experiment (Table 2). The effect of I on SOC and TN was not significant (*P* > 0.05) in 2013, although it was significant (*P* < 0.05) in 2014. In both years, the effect of I on WSC was highly significant (*P* < 0.01), although it was not significant (>0.05) for the SOC:TN ratio. In the CF and OM patterns, there was no significant difference in SOC and TN under the different irrigation treatments in 2013; however, in the I_150_ treatment, SOC and TN were significantly higher than those in the I_0_ treatment in 2014. In both years, under the OM pattern, the WSC in the I_150_ treatment was significantly higher than that in the I_0_ treatment. However, under the NF treatment, there was no significant difference in SOC, TN, the SOC:TN ratio, and WSC between different levels of irrigation. Under the I_150_ treatment, these indices were not significantly different from those under the I_75_ treatment in the 2 years, thereby indicating that excessive brackish groundwater irrigation cannot further increase soil quality.

**Table 2:** Effects of each treatment^a^ on soil organic carbon (SOC), total nitrogen (TN), the C:N ratio, and water-soluble carbon (WSC) in 2013 and 2014^b^.

Year	Fertilize	Irrigation	SOC (g kg^−1^)	TN (g kg^−1^)	SOC: TN ratio	WSC (µg C g^−1^ ds)
2013	CF	I_0_	4.2 ± 0.2cd	0.41 ± 0.03b	10.3 ± 0.7b	119 ± 5ef
		I_75_	4.5 ± 0.3cd	0.45 ± 0.03b	10.0 ± 0.8b	144 ± 10de
		I_150_	5.0 ± 0.4c	0.49 ± 0.04b	10.2 ± 0.7b	152 ± 10cd
	OM	I_0_	6.7 ± 0.7ab	0.64 ± 0.07a	10.4 ± 0.9b	175 ± 21bc
		I_75_	7.4 ± 1.0ab	0.69 ± 0.09a	10.6 ± 0.9b	194 ± 28ab
		I_150_	7.8 ± 1.1a	0.73 ± 0.09a	10.7 ± 1.1b	217 ± 31a
	NF	I_0_	3.8 ± 0.1d	0.30 ± 0.02c	12.7 ± 0.6a	94 ± 2f
		I_75_	4.2 ± 0.1cd	0.32 ± 0.02c	13.0 ± 0.8a	107 ± 5f
		I_150_	4.0 ± 0.2cd	0.31 ± 0.02c	12.9 ± 1.1a	116 ± 7ef
	ANOVA	F	^**^	^**^	^**^	^**^
		I	ns	ns	ns	^**^
		F^*^I	ns	ns	ns	ns
2014	CF	I_0_	4.5 ± 0.3de	0.46 ± 0.04d	9.7 ± 0.7b	153 ± 9cd
		I_75_	5.1 ± 0.4cd	0.52 ± 0.05cd	9.7 ± 0.8b	164 ± 11c
		I_150_	5.5 ± 0.4c	0.58 ± 0.05c	9.5 ± 0.8b	168 ± 12c
	OM	I_0_	7.2 ± 0.7b	0.69 ± 0.06b	10.4 ± 1.0b	218 ± 22b
		I_75_	7.9 ± 0.8ab	0.76 ± 0.08ab	10.4 ± 1.2b	234 ± 23ab
		I_150_	8.2 ± 1.0a	0.81 ± 0.11a	10.1 ± 1.4b	250 ± 31a
	NF	I_0_	4.0 ± 0.2e	0.32 ± 0.02e	12.4 ± 0.8a	124 ± 5d
		I_75_	4.4 ± 0.2de	0.35 ± 0.02e	12.6 ± 0.7a	149 ± 7cd
		I_150_	4.4 ± 0.2de	0.35 ± 0.03e	12.5 ± 0.8a	152 ± 8cd
	ANOVA	F	^**^	^**^	^**^	^**^
		I	^*^	^*^	ns	^**^
		F^*^I	ns	ns	ns	ns

**Notes.**

a CF, Chemical fertilizer; OM, Organic manure and chemical fertilizer; NF, No fertilizer; I_0_, 0 mm irrigation; I_75_, 75 mm irrigation; I_150_, 150 mm irrigation; F, fertilization; I, Irrigation; ANOVA, analysis of variance; ns, Not significant.

b Values are given as means ± standard deviation, and different lowercase letters indicate significant differences at the 5% probability level (LSD; *n* = 3).

* Significant at 5% probability level.

** Significant at 1% probability level.

Under the three irrigation patterns, the SOC, TN, and WSC in the OM treatment were significantly higher than those in the CF and NF treatments. However, the SOC:TN ratio in the CF and OM treatments was significantly lower than that in the NF treatment. The 2-year averages shows that, compared with the NF treatment, the SOC, TN, and WSC in the CF treatment increased by 0.7 g kg^−1^ (16%), 0.2 g kg^−1^ (50%), and 26.2 µg C g^−1^ ds (21%), respectively, and in the OM treatment increased by 3.4 g kg^−1^ (82%), 0.4 g kg^−1^ (122%), and 90.8 µg C g^−1^ ds (73%), respectively. The above data indicate that combined organic manure and chemical fertilizer treatment promoted an increase in soil carbon and nitrogen. The highest amounts of SOC and WSC were recorded in the OM-I_150_ treatment, followed by the OM-I_75_ treatment, whereas the lowest soil carbon pools were observed in the NF-I_0_ treatment.

### Soil microbial biomass carbon and nitrogen (MBC and MBN), the MBC:MBN ratio, soil respiration rate (SR), and metabolic quotient (qCO_2_)

**Figure 2:**
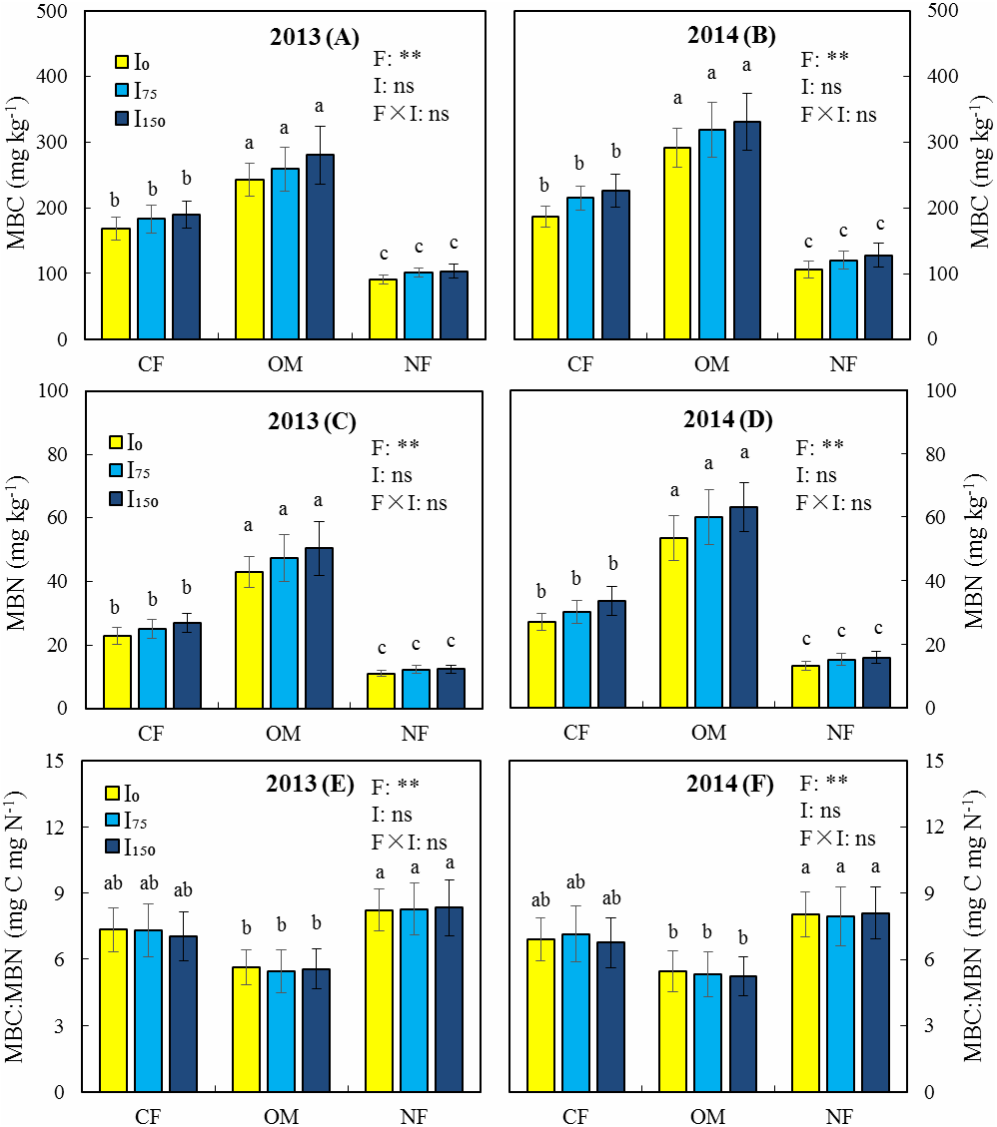
Effects of different treatments on microbial biomass carbon (MBC), microbial biomass nitrogen (MBN), and the MBC: MBN ratio in 2013 and 2014. (A) Microbial biomass carbon in 2013. (B) Microbial biomass carbon in 2014. (C) Microbial biomass nitrogen in 2013. (D) Microbial biomass nitrogen in 2014. (E) MBC: MBN ratio in 2013. (F) MBC: MBN ratio in 2014. Note: CF, Chemical fertilizer; OM, Organic manure and chemical fertilizer; NF, No fertilizer; *I*_0_: 0 mm irrigation; I_75_, 75 mm irrigation; I_150_, 150 mm irrigation. F, fertilization; I, Irrigation; ns, Not significant; *, Significant at 5% probability level; **, Significant at 1% probability level. Vertical bars indicate standard deviation (*n* = 3). Different lowercase letters indicate a significant difference at *p* < 0.05.

**Figure 3:**
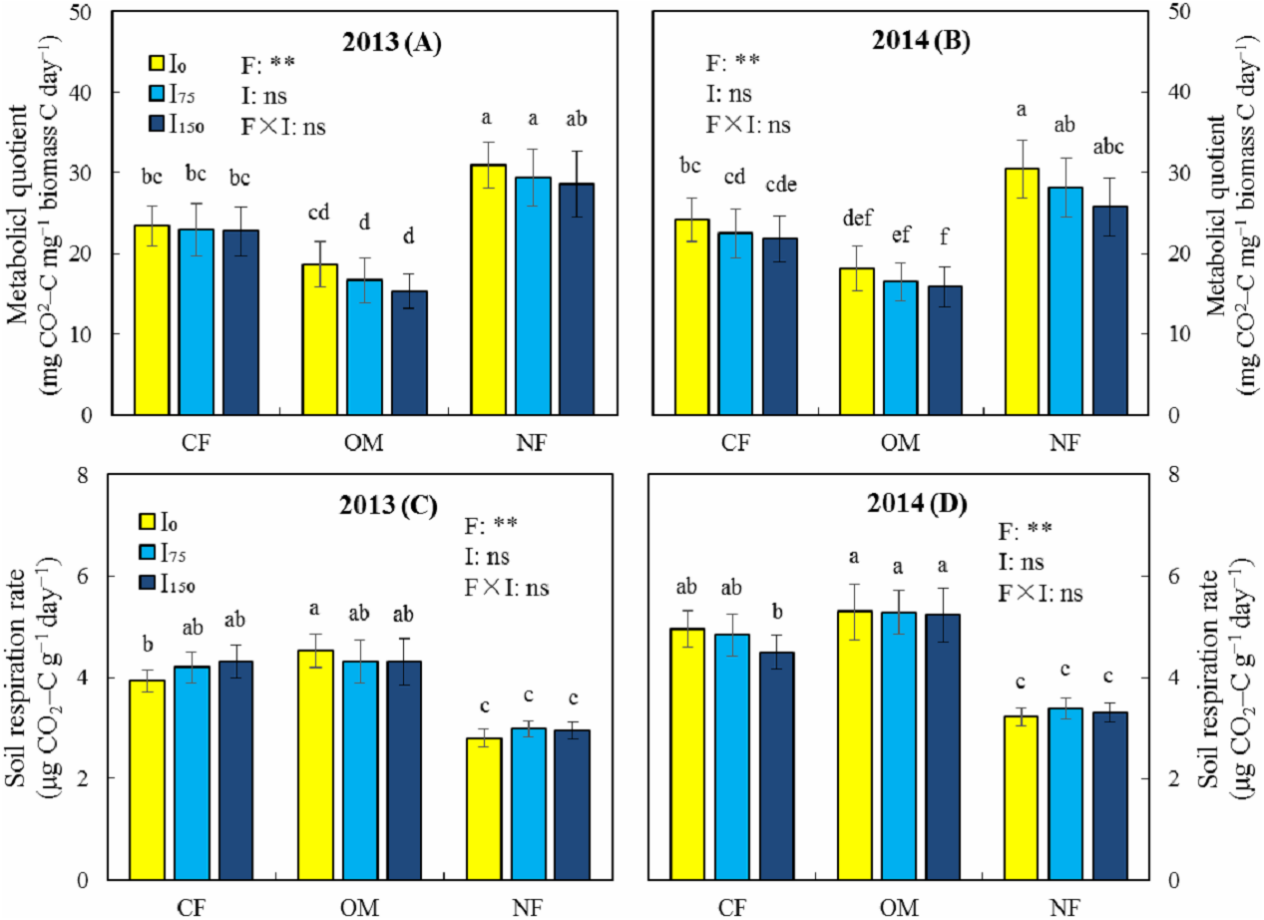
Effects of different treatments on metabolic quotient (qCO_2_) and soil respiration rate in 2013 and 2014. (A) Metabolic quotient in 2013. (B) Metabolic quotient in 2014. (C) Soil respiration rate in 2013. (D) Soil respiration rate in 2014. Note: CF, Chemical fertilizer; OM, Organic manure and chemical fertilizer; NF, No fertilizer; I_0_, 0 mm irrigation; I_75_, 75 mm irrigation; I_150_, 150 mm irrigation. F, fertilization; I, Irrigation; ns, Not significant; * Significant at 5% probability level; ** Significant at 1% probability level. Vertical bars indicate standard deviation (*n* = 3). Different lowercase letters indicate a significant difference at *p* < 0.05.

In both years of the study, variance analysis revealed highly significant effects of F on MBC, MBN, the MBC:MBN ratio, SR, and qCO_2_ (*P* < 0.01) (Figs. 2 and 3), whereas the effect of I on these indicators was not significant (*P* > 0.05). In the three fertilization modes, MBC and MBN did not decrease significantly with an increase in irrigation (Fig. 2), but instead increased slightly. This is due to the low salt content of brackish groundwater and the low irrigation amount of deficit irrigation, which means that the microorganisms were less affected by salt stress. Under the same irrigation level, the MBC and MBN of the OM treatment were significantly higher than those of the CF and NF treatments, whereas the MBC:MBN ratio was significantly lower than that in the NF treatment. This shows that the use of organic manure and chemical fertilizers promotes the accumulation of soil microbial biomass, which is conducive to improving the soil environment. Under irrigation, qCO_2_ in the OM treatment was significantly lower than that in the CF and NF treatments. The OM treatment significantly increased SR compared with the NF treatment, with or without irrigation. The above results show that a combination of organic manure and chemical fertilizer increased SR and reduced qCO_2_, which improved the soil environment more effectively than chemical fertilizer.

### Plant height, leaf chlorophyll relative content (SPAD value), leaf area index (LAI), and biomass

Variance analysis indicated that the effects of F and I on plant height, SPAD value, LAI, and biomass were highly significant (*P* < 0.01) in the two-year experiment (Table 3). Under the three fertilization patterns in the two years, the plant height, SPAD value, LAI, and biomass in the I_150_ treatment were significantly higher than those of the I_0_ treatment, although there was no significant change in these parameters in the I_75_ treatment. The two-year average shows that, compared with the I_0_ treatment, the biomass in the I_75_ and I_150_ treatments increased by 0.9 t ha^−1^ (12%) and 1.6 t ha^−1^ (21%), respectively. The deficit irrigation increased alfalfa plant height, LAI, and leaf SPAD value, thus significantly increasing the biomass.

**Table 3:** Effects of each treatment^a^ on chlorophyll relative content (SPAD value), leaf area index (LAI) and biomass yield in 2013 and 2014^b^.

Year	Fertilize	Irrigation	Plant height (cm)	LAI	SPAD value (%)	Biomass yield (t ha^−1^)
2013	CF	I_0_	45.3 ± 2.1d	3.33 ± 0.19de	39.1 ± 2.1d	7.5 ± 0.3d
		I_75_	47.9 ± 2.4cd	3.63 ± 0.18abc	45.5 ± 2.7c	8.3 ± 0.4bc
		I_150_	50.4 ± 3.1bc	3.79 ± 0.22ab	48.8 ± 3.0bc	8.6 ± 0.6abc
	OM	I_0_	43.3 ± 2.4de	3.39 ± 0.13de	37.7 ± 2.0d	7.9 ± 0.4cd
		I_75_	53.7 ± 3.0ab	3.71 ± 0.16abc	51.0 ± 2.7ab	8.7 ± 0.4ab
		I_150_	55.8 ± 2.6a	3.91 ± 0.22a	54.2 ± 3.5a	9.3 ± 0.5a
	NF	I_0_	40.7 ± 2.0e	3.18 ± 0.17e	31.2 ± 1.6e	6.3 ± 0.4f
		I_75_	44.3 ± 2.0de	3.33 ± 0.16de	34.7 ± 1.7de	6.6 ± 0.3ef
		I_150_	46.7 ± 2.1cd	3.58 ± 0.2bcd	38.1 ± 2.0d	7.1 ± 0.4de
	ANOVA	F	^**^	^**^	^**^	^**^
		I	^**^	^**^	^**^	^**^
		F × I	ns	ns	^*^	ns
2014	CF	I_0_	47.2 ± 2.8de	3.48 ± 0.20cd	42.8 ± 2.2d	8.4 ± 0.5def
		I_75_	51.6 ± 2.8cd	4.02 ± 0.22a	49.1 ± 2.5c	9.3 ± 0.6cd
		I_150_	55.7 ± 3.2bc	4.02 ± 0.22a	51.0 ± 3.1bc	9.8 ± 0.7c
	OM	I_0_	45.9 ± 2.4e	3.37 ± 0.21cd	41.0 ± 2.3de	9.0 ± 0.6cde
		I_75_	60.0 ± 3.2ab	4.10 ± 0.22a	54.0 ± 2.3ab	11.9 ± 0.7ab
		I_150_	68.3 ± 4.7a	4.15 ± 0.21a	57.0 ± 3.7a	12.6 ± 0.8a
	NF	I_0_	42.9 ± 1.9e	3.25 ± 0.20d	33.1 ± 1.9f	6.9 ± 0.5 g
		I_75_	47.2 ± 2.3de	3.52 ± 0.18cd	37.8 ± 1.8e	7.5 ± 0.3fg
		I_150_	51.8 ± 2.1cd	3.63 ± 0.17bc	39.8 ± 1.4de	8.1 ± 0.3ef
	ANOVA	F	^**^	^**^	^**^	^**^
		I	^**^	^**^	^**^	^**^
		F × I	^*^	ns	^*^	^*^

**Notes.**

a CF, Chemical fertilizer; OM, Organic manure and chemical fertilizer; NF, No fertilizer; I_**0**_, 0 mm irrigation; I_75_, 75 mm irrigation; I_150_, 150 mm irrigation; F, fertilization; I, Irrigation; ANOVA, analysis of variance; ns, Not significant.

b Values are given as means ± standard deviation, and different lowercase letters indicate significant differences at the 5% probability level (LSD; *n* = 3).

* Significant at 5% probability level.

** Significant at 1% probability level.

Under the I_75_ and I_150_ treatments in the two years, the plant height and LAI of the OM treatment were significantly higher than those in the NF treatment, although the difference was not significant under the I_0_ treatment. Under the three irrigation modes, the leaf SPAD value and biomass of OM and CF treatments were significantly higher than those in the NF treatment. Compared with the NF treatment, the average biomass in the CF and OM treatments increased by 1.6 t ha^−1^ (18%) and 2.7 t ha^−1^ (31%), respectively. This shows that fertilization increased alfalfa plant height, LAI, leaf SPAD value, and biomass, and the effect of the OM treatment is better than that of the CF treatment. We also found that the interaction of fertilization and irrigation in 2014 had a significant (*P* < 0.05) effect on plant height, leaf SPAD value, and biomass. This indicates that fertilization combined with irrigation is more conducive to the growth of alfalfa. The two-year average shows that the highest biomass (11.0 t ha^−1^) was obtained in the OM-I_150_ treatment, with an increase of 3.4 t ha^−1^ (44.7%) compared with the NF-I_150_ treatment.

## Discussion

Application of organic fertilizers can increase soil organic carbon and promote soil microbial metabolism, thereby increasing soil enzyme activity (Liang et al., 2005). Liu et al. (2010) reported that application of organic fertilizer increased phosphorus-dissolving bacteria in the soil, and therefore urease and alkaline phosphatase activities are increased. Previous studies have also shown that using organic fertilizers increases soil dehydrogenase activity compared with the use of chemical fertilizers (Marinari et al., 2000; Yang et al., 2008). This is due to the fact that organic fertilizers add metabolic substrates that can be consumed by microorganisms, and stimulate dehydrogenase activity (Pascual et al., 1998; Liang et al., 2003). Li et al. (2010) reported that the highest catalase, urease, and phosphatase activities were observed in soils in which both organic and inorganic fertilizers have been used. The results of our study are consistent with the findings of the aforementioned studies; that is, we found that under the combination of organic manure and chemical fertilizers (OM) treatment, the activities of urease, alkaline phosphatase, invertase, catalase, and dehydrogenase were significantly higher than those under the no fertilization and chemical fertilizer treatments (Table 1). Correlation analysis showed that the activities of these five enzymes were significantly (*P* < 0.01) positively correlated with SOC, TN, WSC, MBC, MBN, and SR (Table 4), but negatively correlated with qCO_2_ and the MBC:MBN ratio (*P* < 0.01). This shows that soil enzyme activity is closely related to soil nutrients and microbial biomass.

**Table 4:** Correlation coefficients of soil property parameters with enzymatic activities.

Parameters	Urease	Alkaline phosphatase	Invertase	Catalase	Dehydrogenase
SOC	0.860^**^	0.907^**^	0.696^**^	0.900^**^	0.956^**^
TN	0.943^**^	0.948^**^	0.752^**^	0.934^**^	0.940^**^
SOC: TN ratio	−0.628^**^	−0.362^**^	−0.399^**^	−0.375^**^	−0.170
WSC	0.854^**^	0.942^**^	0.774^**^	0.952^**^	0.918^**^
SR	0.895^**^	0.869^**^	0.879^**^	0.848^**^	0.777^**^
MBC	0.954^**^	0.953^**^	0.799^**^	0.936^**^	0.935^**^
MBN	0.920^**^	0.925^**^	0.785^**^	0.917^**^	0.954^**^
qCO_2_	−0.791^**^	−0.626^**^	−0.501^**^	−0.645^**^	−0.568^**^
MBC: MBN	−0.615^**^	−0.425^**^	−0.444^**^	−0.476^**^	−0.410^**^

**Notes.**

SOC Soil organic carbon TN Total nitrogen WSC Water soluble carbon SR Soil respiration rate MBC Microbial biomass carbon MBN Microbial biomass nitrogen qCO_2_ Metabolic quotient

* Significant at the 0.05 probability level (*n* = 9).

** Significant at the 0.01 probability level (*n* = 9).

Soils with high SOC content have a more stable aggregate structure that increases the infiltration and retention of moisture and is less susceptible to erosion and runoff (Watts et al., 2006; Abiven, Menasseri & Chenu, 2009). Ebhin Masto et al. (2006) reported that the combination of organic and chemical fertilizers significantly increased SOC compared with chemical fertilizer alone. Similarly, Bhandari et al. (2002) reported that the combed application of organic and chemical fertilizers will delay the release of nitrogen in soil, resulting in less nitrogen loss and thus higher TN. Combinations of organic fertilizers and chemical fertilizers are more effective measures for increasing soil fertility and improving the soil environment than are applications of single chemical fertilizers (Hopkins & Shiel, 1996; Bharambe & Tomar, 2004). Our results are again consistent with the aforementioned studies, in that the OM treatment significantly increased SOC, TN, and WSC compared with the CF treatment, reducing the SOC:TN ratio (Table 2) and thus improving soil quality better than CF.

Previous studies have shown that a reduction in MBC is often due to a lack of phosphorus, potassium, or other micronutrients in the soil (Moharana et al., 2012). The use of organic fertilizers not only increases phosphorus and potassium in the soil but also introduces a large amount of micronutrients and metabolic substrates, thereby promoting the growth of microorganisms and increasing the MBC (Luo & Sun, 1994; Hao et al., 2008). Organic fertilizers also provide a stable source of organic carbon and nitrogen for the growth of soil microorganisms (Marschner, Kandeler & Marschner, 2003; Mandal et al., 2007), thereby increasing MBC and MBN (Mandal et al., 2007). Our results are consistent with these findings, as we demonstrated that a combination of organic and chemical fertilizers increase SOC, TN, and WSC (Table 2), and thus significantly increased MBC and MBN. The metabolic quotient (qCO_2_) is the ratio of SR to MBC, which reflects microbial metabolic activity and is also a valid indicator of soil ecosystem health (Anderson & Domsch, 1993). A lower qCO_2_ indicates a higher substrate mass and microbial metabolic efficiency in the soil (Anderson & Domsch, 1990). Previous studies have shown that a lower qCO_2_ was observed in soils with organic fertilizer application (Dilly, 2005; Melero et al., 2007; Scheller & Joergensen, 2008). Consistently, we also observed a lower qCO_2_ under the OM treatment than under the CF and NF treatments (Fig. 3), indicating that the OM treatment increases substrate quality and microbial metabolic efficiency in soil. We also found that the OM treatment significantly increased SR compared with the NF treatment. This is consistent with the reports of Liang et al. (2005) and Liang et al. (2003), who found that organic fertilizers not only increase the activity of various enzymes but also increase the SR.

Previous studies have shown that the activities of most soil enzymes, such as invertase, polyphenol oxidase, and catalase, are obviously decreased after brackish water irrigation (Guo, 2012), for the following three reasons: (a) Brackish water irrigation increases the amount of salt in the soil, exposing soil microorganisms to osmotic stress, and thereby inhibiting microbial metabolism (Rietz & Haynes, 2003); (b) Salinity in the soil inhibits the dissolution of proteins and destroys the tertiary structure of the proteins, resulting in the denaturation and inactivation of proteases, and thereby affecting enzyme activity (Yuan et al., 2007); (c) Salinity in the soil suppresses the growth of crop root systems, thereby reducing the root enzyme secretion (Ghollarata & Raiesi, 2007). Wang et al. (2009) reported that the use of brackish groundwater irrigation causes an excessive accumulation of salt in the soil and increases in soil pH, thereby significantly reducing soil urease, invertase, and catalase activities. However, alkaline phosphatase activity increased with an increase in brackish water. Similarly, we found that irrigating with 150 mm brackish groundwater per year (I_150_) significantly reduced invertase activity and significantly increased alkaline phosphatase activity compared with no irrigation (Table 1). Singh, Chauhan & Minhas (2009) also found that low concentrations of NaCl (<20 mmol L^−1^) have a beneficial effect on alkaline phosphatase, and Li et al. (2010) reported that deficit irrigation can increase the activities of soil catalase, urease, and invertase to improve the soil environment. Our study showed that the I_150_ treatment significantly increased soil urease and catalase activities under the OM fertilization mode compared with no irrigation; however, irrigation with brackish groundwater did not significantly increase both enzyme activities without fertilization. This is due to the fact that the combination of organic fertilizer and chemical fertilizer can alleviate the effect of salt stress on soil enzyme activity. In addition, the brackish groundwater in our study area has a low salinity (NaCl: 2.3 g L^−1^) and the irrigation amount (150 mm) of the I_150_ treatment is also lower than that of the conventional irrigation in the area (300 mm). Therefore, enzyme activities under the OM-I_150_ treatment were less inhibited by salt stress, and indeed, the highest urease, alkaline phosphatase, catalase, and dehydrogenase activities were observed under this treatment.

Previous studies have shown that long-term brackish groundwater irrigation resulted in decreases of 26.8% and 28.0% for organic matter and total nitrogen, respectively (Wang, Zhao & Ouyang, 2010). They also found that brackish groundwater irrigation inhibited soil microbes, reducing MBC and MBN by 24.4% and 42.4%, respectively. Wichern, Wichern & Joergensen (2006) reported that low concentrations of NaCl have a stimulating effect on soil microorganisms, whereas high concentrations of NaCl are not conducive to the growth and reproduction of microorganisms and result in reduced enzyme activity. Our results show that irrigation has insignificant effects on MBC and MBN (Fig. 2). This is due to the lower salt content of the brackish groundwater we used combined with the use of an irrigation volume that is lower than the conventional irrigation practice in the area, and therefore irrigation has less effect on the microorganisms. In addition, we found that MBC and MBN increased slightly with an increasing irrigation volume (Fig. 2), which is similar to the results of Shui et al. (2012), who found that deficit irrigation increased maize yield and soil MBC. It has been reported that excessive irrigation can increase evapotranspiration, wheat dry matter, and leaf area per plant, but it does not significantly increase grain yield and reduces the soil respiration rate (SR) (Kang, Chen & Wan, 2010). Soil respiration is mainly attributable to root respiration and microbial oxidation of organic matter, and is an indicator of soil quality (Rong et al., 2015). Previous research results have revealed that SR improved with increasing irrigation under deficit irrigation, but that overirrigation inhibited SR (Wang, Zhao & Ouyang, 2010). Our results show that deficit irrigation did not significantly increase or decrease SR (Fig. 3), which could be attributable to the combination of lower irrigation volume and brackish groundwater salt stress, resulting in a small change in SR. Triantafilis et al. (2004) showed that irrigation with low-salinity brackish water did not affect crop yields, whereas high concentrations of brackish water were detrimental to crop growth. Talebnejad & Sepaskhah (2015) also reported that irrigating with saltwater below 20 dS m^−1^ conductivity did not reduce the biomass of quinoa, whereas Zhang (1999) found that saltwater irrigation with less than 3 g L^−1^ of NaCl promoted the growth of maize seedlings to varying degrees, although more than 3 g L^−1^ was detrimental to crop growth.

Previously, Singh, Chauhan & Minhas (2009) showed that winter wheat yield increased with brackish water irrigation. In the present study, we showed that irrigation with low saline (NaCl: 2.3 g L^−1^) brackish groundwater increased alfalfa plant height, LAI, and leaf SPAD values, thereby increasing biomass yield (Table 3). In arid and semi-arid regions, moderate amounts of brackish groundwater provide good water conditions for alfalfa growth. Although brackish groundwater contains some salt, the salt concentration in the soil is lower due to the smaller irrigation volume. Therefore, salt stress had a less inhibitory effect on alfalfa growth. In contrast, under the OM treatment, the use of brackish groundwater for deficit irrigation is more conducive to alfalfa growth. This is due to the significant increase in MBC and MBN under the OM treatment (Fig. 2). This also increased soil enzyme activity and soil carbon and nitrogen contents (Tables 1 and 2), thus providing more nutrients and a good soil environment for alfalfa growth. Finally, we observed the highest alfalfa biomass yield (11.0 t ha^−1^) under the OM-I_150_ treatment.

## Conclusion

The application of organic manure and chemical fertilizers (OM) significantly increased soil urease, alkaline phosphatase, invertase, catalase and dehydrogenase activities, and also increased the SOC, WSC, TN, MBC, MBN and SR, but decreased the SOC:TN ratio and qCO_2_, thus improving the soil quality in abandoned farmland. Under the OM treatment, the use of brackish groundwater with low salt concentration increased the activity of urease, alkaline phosphatase, catalase, and dehydrogenase, which is beneficial to alfalfa growth and increases biomass yield. We observed the highest alkaline phosphatase, urease, catalase and dehydrogenase enzyme activities, as well as the highest SOC, TN, WSC, MBC, MBN, and alfalfa biomass yield under the OM-I_150_ treatment. Therefore, the OM-I_150_ treatment could be used as an effective measure to improve the soil quality of abandoned farmland and to make rational use of brackish groundwater in the arid and semi-arid regions of northern China.

##  Supplemental Information

10.7717/peerj.4410/supp-1 Data S1 Raw data

## Additional Information and Declarations

### Data Availability

The following information was supplied regarding data availability: The raw data is provided as Data S1.
